# Draft Genome Sequence of a Urinary Isolate of *Lactobacillus crispatus*

**DOI:** 10.1128/genomeA.01278-16

**Published:** 2016-12-01

**Authors:** Travis K. Price, Majed Shaheen, Laurynas Kalesinskas, Kema Malki, Evann E. Hilt, Catherine Putonti, Alan J. Wolfe

**Affiliations:** a Health Sciences Division, Department of Microbiology and Immunology, Stritch School of Medicine, Loyola University Chicago, Maywood, Illinois, USA; b Bioinformatics Program, Loyola University Chicago, Chicago, Illinois, USA; c Department of Biology, Loyola University Chicago, Chicago, Illinois, USA; d Department of Computer Science, Loyola University Chicago, Chicago, Illinois, USA

## Abstract

While *Lactobacillus crispatus* contributes to the stability of normal vaginal microbiota, its role in urinary health remains unclear. As part of an on-going attempt to characterize the female urinary microbiota, we report the genome sequence of an *L. crispatus* strain isolated from a woman displaying no lower urinary tract symptoms.

## GENOME ANNOUNCEMENT

As part of an attempt to characterize the newly discovered female urinary microbiota (1–8), we report the genome sequence and annotation of a strain of *Lactobacillus crispatus* isolated from the bladder of an adult female. This is the first genome report of a urinary isolate of *L. crispatus*, a species associated with bladder health (8).

Using the expanded spectrum version (9) of the enhanced quantitative urine culture protocol (2), *L. crispatus* strain C037 was isolated from a healthy female not displaying any urinary symptoms. The strain was subcultured to purity, analyzed by matrix-assisted laser desorption/ionization-time-of-flight mass spectrometry, and pure cultures were stored at −80°C in 2 mL CryoSaver Brucella broth with 10% glycerol, no beads, cryovials (Hardy Diagnostics). For genome extraction, the preserved pure culture isolate was grown on 5% sheep blood agar (BDBBL™ prepared plated media) under 5% CO_2_ at 35°C for 24 h.

To extract genomic DNA, cells were resuspended in 0.5 mL DNA extraction buffer (20 mM tris-Cl, 2 mM EDTA, 1.2% Triton X-100, pH 8) followed by addition of 50 µL lysozyme (20 mg/mL), 30 µL mutanolysin, and 5 µL RNase (10 mg/mL). After a 1-h incubation at 37°C, 80 µL 10% SDS and 20 µL proteinase K were added followed by a 2-h incubation at 55°C. Then, 210 µL of 6M NaCl and 700 µL phenol-chloroform were added. After a 30-min incubation with rotation, the solution was centrifuged at 13,500 rpm for 10 min, and the aqueous phase extracted. An equivalent volume of isopropanol was added; after a 10-min incubation, the solution was centrifuged at 13,500 rpm for 10 min. The supernatant was decanted and the DNA pellet precipitated using 600 µL 70% ethanol. Following ethanol evaporation, the DNA pellet was resuspended in tris-EDTA and stored at −20°C.

Genomic DNA was diluted in water to a concentration of 0.2 ng/µl. Library preparation was performed using the Nextera XT DNA library preparation kit (Illumina) according to manufacturer’s instructions with 1 ng of input DNA. The isolate was sequenced twice, on two separate runs, using the Illumina MiSeq platform and the MiSeq reagent kit v2 (300-cycles). Sequence assembly was performed using Velvet (10) (*k* = 99) followed by SSPACE (11) for scaffolding. *L. crispatus* C037 was assembled into 96 scaffolds with a genome coverage of 113×. The scaffolds include 2.147 Mbp of sequence with a G+C content of 36.6%. Gene annotations were performed using GLIMMER (12) and tRNA-Scan (13) identifying 2,096 protein coding genes, 65 RNA (tRNA and rRNA) genes, and four clustered regularly interspaced short palindromic repeats (CRISPR) (14). The 16S rRNA gene sequence of the urinary isolate C037 was identical to that of the species’ type strain *L. crispatus* ST1 (NR_074986), an avian enteric strain. One scaffold (10,704 bp in length) produced a hit to the 16,663 bp *L. crispatus* plasmid pLc17 (KR052811); while a significant proportion of the plasmid sequence was detected, a complete, circularized assembly was not possible.

### Accession number(s).

The draft whole-genome project for *L. crispatus* C037 has been deposited at DDBJ/EMBL/GenBank under accession number MAKH00000000.
